# Selection for Adaptation to Dietary Shifts: Towards Sustainable Breeding of Carnivorous Fish

**DOI:** 10.1371/journal.pone.0044898

**Published:** 2012-09-28

**Authors:** Richard Le Boucher, Mathilde Dupont-Nivet, Marc Vandeputte, Thierry Kerneïs, Lionel Goardon, Laurent Labbé, Béatrice Chatain, Marie Josée Bothaire, Laurence Larroquet, Françoise Médale, Edwige Quillet

**Affiliations:** 1 INRA, UMR1313 GABI Génétique Animale et Biologie Intégrative, Jouy-en-Josas, France; 2 Ifremer, UMR110 Intrepid, Palavas-les-Flots, France; 3 AgroParisTech, UMR1313 GABI, Paris, France; 4 INRA, UE 937 PEIMA, Sizun, France; 5 INRA, UMR1067 NuMeA Nutrition, Metabolism, Aquaculture, St Pée-sur-Nivelle, France; The University of Plymouth, United Kingdom

## Abstract

Genetic adaptation to dietary environments is a key process in the evolution of natural populations and is of great interest in animal breeding. In fish farming, the use of fish meal and fish oil has been widely challenged, leading to the rapidly increasing use of plant-based products in feed. However, high substitution rates impair fish health and growth in carnivorous species. We demonstrated that survival rate, mean body weight and biomass can be improved in rainbow trout (*Oncorhynchus mykiss*) after a single generation of selection for the ability to adapt to a totally plant-based diet (15.1%, 35.3% and 54.4%, respectively). Individual variability in the ability to adapt to major diet changes can be effectively used to promote fish welfare and a more sustainable aquaculture.

## Introduction

In fish, as in other vertebrates, within-species or within-population diversity of individual foraging strategies is a common feature [1], [2], [3]. Both phenotypic plasticity [3] and genetic variability probably contribute to this diversity. Besides, there is growing evidence that changes in feeding can drive evolution, as demonstrated in stickleback [4], lizards [5] or in human sub-populations [6], [7], [8], [9]. Evidence of rapid dietary-induced genetic changes can also be seen from within-population experimental selections in animal models [10], [11]. Selective breeding of carnivorous fish for aquaculture may take advantage of this plasticity to address an especially challenging feeding context by promoting such genetic adaptation in farmed fish populations fed with non-marine diets.

Providing high quality food to an ever-increasing human population in a sustainable way is clearly the challenge of this century, and aquaculture has been identified as a significant but incomplete solution [12], [13]. Between 2002 and 2006, the earth became home to 300 million more human beings, while consumption of aquatic products rose from 100.7 to 110.4 million tons [14]. Since global fish capture for food plateaued at around 60 million tons during the 1980s, this increase has been entirely covered by aquaculture, and fish production for human consumption is still expected to rise by 1.2 to 1.5 million tons per year [14]. Despite an uneven development in different areas, world farmed fish production is still rapidly growing for both marine (+7.2% per year) and freshwater (+11.9% per year) fish [14]. However, aquaculture heavily relies on the use of fishmeal and fish oil in feed, whereas fisheries that provide these feedstuffs have reached their limit and will not be able to meet increasing needs [15]. The main response to the limitation of these essential feedstuffs has been the increased substitution of fishmeal and fish oil with terrestrial plant-based products [15], especially in Europe where most animal by-products are prohibited in animal feed. For example, the share of fish meal and fish oil in salmon diets decreased from 45% to 30% between 1995 and 2006 [15] and is now close to 20% in commercial diets. However, higher substitution rates strongly reduce fish growth [16], [17] and have an impact on health [16], [18], which is a major obstacle to the aquaculture development of many species that still rely on marine ingredients such as salmonids and marine species. Interestingly, genetic variability of the ability to grow on a plant-based diet has recently been demonstrated in farmed fish populations of two carnivorous species, the rainbow trout [19], [20], [21] and the European whitefish [22], but the effectiveness of selective breeding for this specific trait has only been tested with a fishmeal-free diet [23]. The objectives of this study were to show that fish can actually be selected for their ability to adapt to a diet totally free of marine ingredients and to characterize the effects of such a selection scheme on major production traits.

## Materials and Methods

### Ethical statements

Under French regulation, the INRA facilities are authorized for animal experimentation (B 29–277–02). Technical staff and scientists have personal authorizations to conduct animal experimentations in accordance with good animal practice delivered by the DDPP (Service de Protection et de Surveillance Sanitaire des Animaux et des Végétaux).

### Base population

The experimental lines were issued from the INRA-Synthetic strain (INRA-SY) of rainbow trout, *Oncorhynchus mykiss*, a domesticated population assumed to have a large genetic variability, maintained without any selection since the early 1980s and fed with standard commercial diets.

### Selected and control breeders

Fish were issued from a full factorial cross among SY parents (36 dams ×44 sires, *i.e.*, 1584 full-sib families). Young fish were reared for 1 year in flow-through tanks in INRA experimental facilities (PEIMA, Sizun, France) and fed a diet totally devoid of marine products (PB1, Table 1, Table S1) since the first meal. Sequential upward selection was applied by sorting for body size at 95, 203, 329, 360 days post-fertilization (dpf) and approximately half of the fish were discarded each time. The overall selection pressure was 3.25%. Selected fish (S) were the largest ones among those that survived up to the different culling times. Sixty fish (30 dams and 30 sires) were then randomly sampled among the sexually mature S fish as breeders for the next generation. Non-selected control (C) breeders (30 sires and 30 dams) were also randomly sampled among a batch of the original SY strain fed with a commercial diet containing fish meal and fish oil. At that time, no data was available on the effects of any plant-based diet on the reproductive performance of trout. Thus, the diet of selected fish (S) was shifted to the same commercial diet (370 g) as C breeders (Le Gouessant, France) in order to prevent any defect of gamete quality potentially associated with the PB diet. This aimed at reducing potential sources of differences in gamete quality between S and C breeders, and minimizing non genetic parental effects known to affect growth traits in early stages [24].

**Table 1:** Ingredients and proximal composition of the experimental diets M (M1, M2) and PB (PB1, PB2).

Diets	M1^1^	M2^1^	PB1^2^	PB2^2^
*Ingredients (g/kg)*				
Fishmeal	692	623	0	0
Maize gluten	0	0	250	170
Soybean meal	0	0	208	200
Wheat gluten	0	0	239	250
Extruded wheat	187	240	0	50
White lupin	0	0	70	57
Extruded dehulled pea	0	0	0	30
Fish oil	81	97	0	0
Rapeseed oil	0	0	62	62
Linseed oil	0	0	37	37
Palm oil	0	0	24	24
Soya lecithin	0	0	20	20
L-Lysine	0	0	15	15
L-Arginine	0	0	0	10
CaHPO4.2H20 (18%P)	0	0	35	35
Binder	20	20	20	20
Min. Premix	10	10	10	10
Vit. Premix	10	10	10	10
*Composition*				
Crude Protein (% dry matter)	54.6	47.1	50.5	44.8
Lipids (% dry matter)	15.6	22.8	16.2	23.3
Energy (kJ/g dry matter)	22.5	23.5	23.2	23.6

1 M1 diet was given from first feeding to 236 dpf and M2 from 236 dpf until the end of the trial to adapt protein and lipid content to the nutritional requirements of larger fish.

2 PB1 diet was given from first feeding to 280 dpf and PB2 from 280 dpf until the end of the trial to adapt protein and lipid content to the nutritional requirements of larger fish.

### Experimental crosses

To evaluate the response to selection, two crosses (full factorial between sires and dams) were carried out, mating the S and the C parents, respectively, in order to obtain one batch of 3600 S eggs and one batch of 3600 C eggs. After hatching, half of each batch was fed a plant-based (PB) diet (S-PB and C-PB groups, respectively) to assess selection gain for survival and growth on the PB diet. The other half of each batch was fed a marine (M) diet (S-M and C-M groups, respectively) to assess the non-specific selection gain. For each of the four line x diet combinations, three rearing units (three tanks seeded with 600 eggs) were used.

Two additional crosses were implemented in order to check possible non genetic maternal effects which may bias the estimates of the direct genetic gain from the comparison of pure lines, especially at early stages. Breeders were the same as previously mentioned. The same day, two crosses were produced (full factorial between sires and dams), mating S sires with C dams to produce the SC cross and C sires with S dams to produce the CS cross. The two crosses were managed the same way as pure lines, *i.e.* 3600 eggs were randomly sampled in each cross and divided in two groups. Swim-up fry from these groups were fed either the plant-based diet (respectively SC-PB and CS-PB groups) or the marine diet (respectively SC-M and CS-M groups) and reared in triplicates (three tanks 600 fish each).

### Experimental diets

The major ingredients in the M diet were fish meal and fish oil, and the PB diet was totally devoid of marine products that were replaced by a blend of plant products (Table 1). Experimental diets were produced in INRA facilities (Donzacq, France) and distributed as of the first meal (41 dpf). They were formulated to be isoproteic, isolipidic and isoenergetic, and the composition was changed at 236 dpf for M diet (M1 diet to M2 diet) and at 280 dpf for PB diet (PB1 diet to PB2 diet) to adapt protein and lipid content to the nutritional requirements according to fish size [25] (Table 1, Table S1). Product sources in the PB diet were chosen to satisfy trout requirements in fatty acids, amino acids, vitamins and minerals [25]. Soy lecithin, lysine and calcium phosphate were added to the PB diet to adjust the phospholipid, essential amino acid and available phosphorus contents. Food was provided to satiation. Feeding rates were determined according to feeding tables established for rainbow trout in the PEIMA facility. Rations were adjusted to every tank biomass and upped by 30% to ensure food was not limiting in any of the tanks during the trial.

### Data collection

Each tank was seeded with 600 eyed eggs (18 dpf). Surviving fish at first feeding (41 dpf) were estimated from the number of dead fish (removed daily) from 18 dpf to 41 dpf. At this stage, survival rate was similar in all tanks (93.8%, P>0.05). Later on, fish were individually counted at 60 dpf, 145 dpf, 193 dpf and 291 dpf, and survival (*Su*) was calculated based on number of surviving fish at 41 dpf. Throughout the trial, water characteristics (temperature, pH, NH_3_, NO_2_) were surveyed. After 145 dpf, data were not collected in additional crosses (CS and SC).

Multiple random samplings (at least 5 per sampling date) of 50 fish per tank were performed about once a month during the first year of life (60 dpf, 102 dpf, 145 dpf, 168 dpf, 193 dpf, 216 dpf, 236 dpf, 291 dpf, 312 dpf, 355 dpf) to estimate the mean body weight (*BW* in grams) in each of the 12 experimental tanks. At 145 dpf, 193 dpf and 291 dpf, within tank biomass was estimated (*Biom*  =  Number of fish x *BW*).

At 145 dpf, 193 dpf and 291 dpf, individual body weights were also recorded on more than 100 fish per tank to estimate the within tank coefficient of variation (*CV*: standard deviation/mean, in %).

### Whole body proximate composition and fatty acid content

At 291 dpf, two samples of 5 fish were taken in each tank of S and C fish. They were weighed, frozen and stored at −20°C pending analysis. Chemical composition and lipid analysis were performed on whole fish samples (2 pools of 5 fish per tank) and experimental diets. Dry matter content (DM, in %) was measured after drying at 105°C for 24 h and protein content (PT, in % of wet weight) by the Kjeldahl method. Total lipids were extracted [26] and fatty acid compositions (FA, in mg.g lipid^−1^) were determined on the total lipid extract (Lip, in % of wet weight) with prior preparation of methyl-esther. Extraction, purification and identification using gas chromatography were previously described [27]. Gross energy (Ener, in % of wet weight) of the samples was measured after combustion in an adiabatic bomb calorimeter.

### Statistical analyzes

*BW*, Biom, *CV* and *Su* means were used to assess the significance of diet and line effects as well as interaction between diet and line. All effects were tested with the mixed model (1) (SAS-MIXED, SAS Institute Inc., Cary, NC).
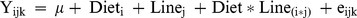
(1)Where Y_ijk_ is the performance of the tank k, µ is the general mean, Diet_i_ is the fixed effect of the diet i (M *vs*. PB), Line_j_ is the fixed effect of the Line j (S, C or S, C, SC, CS), Diet*Line_(i*j)_ is the interaction between Diet i and Line j and e_ijk_ is the random residual which corresponds to the tank effect.

Survival rates (%) were previously arcsine-root transformed. To analyse the line effect, *BW* and *Biom* were log-transformed as fish *BW* were largely heavier in M batches. For chemical composition traits (DM, PT, Lip, Ener, FA), analyses were duplicated and replicate effect was added into the model. Preliminary analyses showed that FA were influenced by the weight of the fish and they were therefore analyzed with *BW* as a covariate.

### Response to selection

Three different gains were estimated for the three traits of interest (*Su*, *BW* and *Biom*).

The direct selection gain for trait X with the PB diet:
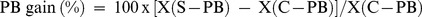
(2)


The direct selection gain for trait X observed with the M diet, *i.e.* the non-specific response to selection:
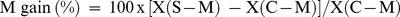
(3)


The PB-specific gain for trait X, *i.e.* the specific response to selection for ability to perform when fed the PB diet:

PB-specific gain (%)  =  PB gain – M gain

## Results and Discussion

**Figure 1:**
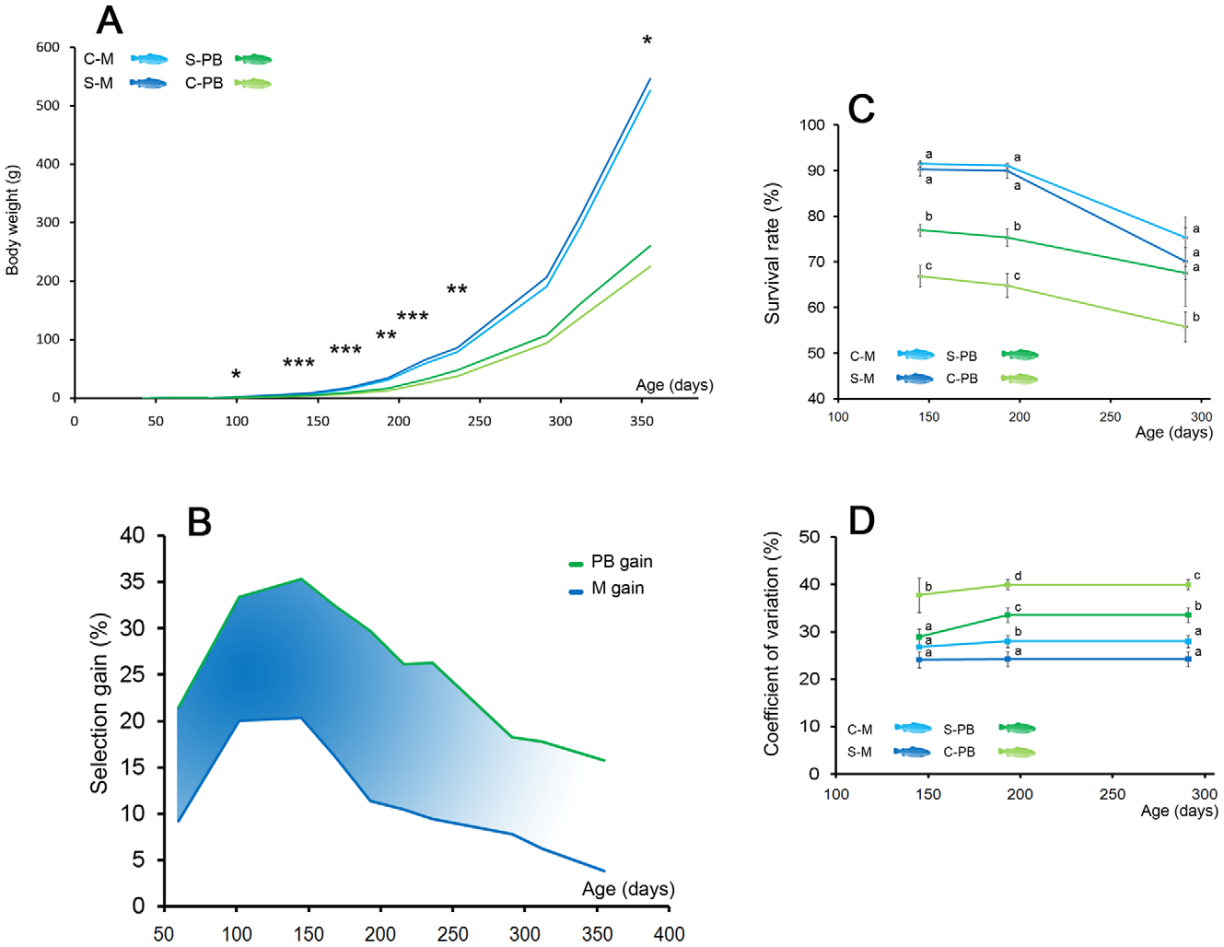
Effects of selection on survival and growth performances. (A) Ten successive weight measurements were carried out during the first year of life. Growth curves are shown for the four line x diet combinations. Stars denote the significance of the diet*line interaction for body weight at every date of measurement (*: P<0.1; **: P<0.05; ***: P<0.01). (B) Selection gain for body weight (%) is calculated for fish fed with a plant-based (PB) diet (green line) and fish fed with a marine diet (blue line). The difference between the two gains is the PB diet-specific selection gain for the ability to grow when fed with a plant-based diet after one generation of selection. (C) The survival rate (%) was significantly higher in selected fish when fed PB diet when there was no difference between selected and unselected population when fed M diet. Different letters mean significant differences (P<0.05) between groups. (D) The coefficient of variation of body weight (%) was higher in groups of fish fed the PB diets throughout the trial, but was significantly reduced by selection in the case of the PB diet. Different letters mean significant differences (P<0.05) between groups.

High substitution of marine ingredients with plant-based products [18] is usually known to have negative effect on fish survival rate. The analysis of survival in the experimental groups confirmed that the PB diet had a deleterious effect on survival as of the early stages of development (91.5% and 90.3% mean survival in M groups *vs*. 66.9% and 77.0% in PB groups at 145 dpf) (Fig. 1C and Table 2). However, in the case of the PB diet, mean survival rate was substantially improved by selection since very early stages following first meal (77.0% in S-PB offspring vs. 66.9% in C-PB offspring after 145 dpf; P<0.01), whereas no difference was recorded in the case of the M diet. During the summer period, some mortalities attributed to a temporary degradation of the inlet water quality were recorded. They were higher in M groups (their larger size was associated to higher biomass, kg/m3).

**Table 2:** Ls-means of body weight (*BW*), survival rate (*Su*), biomass (*Biom*) and coefficient of variation (*CV*) for ten dates and their statistical analyzes for line, diet effects and line*diet interaction.

Trait^6^	Dpf^5^	C-M	C-PB	S-M	S-PB	Diet	Line	Diet*Line
***BW*** 1	60	0.3±0.0	0.2±0.0	0.4±0.0	0.2±0.0	0.0001	0.0001	0.2131
***BW*** 1	102	2.0±0.0	0.9±0.0	2.4±0.0	1.2±0.0	0.0001	0.0001	0.0791
***BW*** 1	145	6.9±0.1	3.4±0.1	8.3±0.1	4.6±0.1	0.0001	0.0001	0.0098
***BW*** 1	168	14.8±0.2	7.1±0.2	17.2±0.2	9.4±0.2	0.0001	0.0001	0.0014
***BW*** 1	193	30.9±0.5	12.8±0.5	34.4±0.5	16.6±0.5	0.0005	0.0001	0.0482
***BW*** 1	216	59.3±0.9	25.3±0.9	65.5±0.9	31.9±0.9	0.0001	0.0001	0.0097
***BW*** 1	236	78.8±2.3	37.7±2.3	86.2±2.3	47.6±2.3	0.0006	0.0001	0.0377
***BW*** 1	291	188.8±5.4	91.0±5.4	203.5±5.4	107.6±5.4	0.0033	0.0001	0.1588
***BW*** 1	312	292.2±7.8	137.2±7.8	310.4±7.9	161.6±7.8	0.0040	0.0001	0.1106
***BW*** 1	355	526.2±10.9	225.1±10.9	546.3±10.9	260.5±10.9	0.0107	0.0001	0.0898
***Su*** 2	145	91.5±0.9	66.9±0.9	90.3±0.9	77.0±0.9	0.0001	0.0027	0.0003
***Su*** 2	193	91.1±1.1	64.9±1.1	90.0±1.1	75.3±1.1	0.0001	0.0098	0.0019
***Su*** 2	291	75.4±3.1	55.8±3.1	70.1±3.1	67.6±3.1	0.0064	0.3397	0.0254
***Biom*** 3	145	3.5±0.0	1.3±0.0	4.1±0.0	2.0±0.0	0.0001	0.0001	0.0008
***Biom*** 3	193	15.6±0.2	4.6±0.2	17.1±0.2	6.9±0.2	0.0001	0.0001	0.0007
***Biom*** 3	291	78.7±4.6	28.1±4.6	79.1±4.6	40.2±4.6	0.0001	0.0202	0.0190
***CV*** 4	145	26.8±1.3	37.8±1.3	24.1±1.3	28.9±1.3	0.0022	0.0003	0.0460
***CV*** 4	193	28.0±0.8	40.0±0.8	24.2±0.8	33.6±0.8	0.0002	0.0001	0.1374
***CV*** 4	291	25.9±1.9	42.5±1.9	23.5±1.9	35.4±1.9	0.0338	0.0001	0.2497

1 Body weight in grams.

2 Survival rate in %.

3 Biomass in kg.

4 Coefficient of variation of *BW* in %.

5 Dates in days post-fertilization.

6 Standard-errors are indicated after each value as well as significance of diet effect (Diet), line effect (Line) and the interaction between diet and line (Diet*Line).

The nutrient content of both diets were very similar (Table 1, Table S1), but the PB diet did not contain any n−3 HUFA (highly unsaturated fatty acids), eicosapentaenoic acid (20:5n−3, EPA) or docosahexaenoic acid (22:6n−3, DHA) (Table S1), that favor the growth of healthy fish [16], [18]. The observed gain in survival in S group may be the result of a better ability of the fish to cope with this deficiency in the early stages or of a lesser sensitivity to anti-nutritional factors contributed by plant protein sources. However, more studies are necessary before any conclusions on the physiological mechanisms of adaptation can be drawn.

The increase of body weight with age in control and selected groups fed PB or M diets is presented in Fig. 1A. There was a strong effect of the diet, with M fish being twice as large as PB fish as of 60 dpf (Table 2). The interaction between diet and selection effects estimated in a SAS-MIXED model was used as an indicator of the efficiency of the selection for specific adaptation to the PB diet. It was significant as of 145 dpf (P<0.01) and remained significant until 291 dpf (Fig. 1A and Table 2). The PB gain for body weight increased from 21.4% (60 dpf) to 35.3% (145 dpf) and then decreased to 15.7% (355 dpf). The M gain increased from 9.2% (60 dpf) to 20.3% (145 dpf) and then decreased to 3.8% (355 dpf) (Fig. 1B). This latter gain is due to the individual selection for overall growth performance but is not linked to the specific ability to grow with the PB diet. To take this non-specific gain into account, the PB-specific response to selection for body weight was calculated as the difference between the previous ratios (blue area in Fig. 1B). Figure 1B shows that it was around 12% and remained quite stable throughout the first year of life when specific gains (M and PB) reached a maximum between 100 and 145 dpf, which corresponds to the age of the first and the most significant (50%) culling of the selected breeders.

The survey of the additional crosses (SC-M, SC-PB, CS-M and CS-PB) showed that there were no significant differences (model (1), P>0.05) at 145 dpf between the CS and SC crosses for *Su*, *BW* and *Biom* (Table S2). This confirmed that this difference between selected and control fish was not the consequence of maternal effects associated to C or S females and could be attributed to selection.

These results demonstrate for the first time that at least two major production traits (survival and growth) associated with the ability to adapt to plant-based feedstuff were positively modified after a single generation of selection, indicating that domesticated populations of rainbow trout have the genetic potential to adapt to major dietary changes.

A higher heritability for *BW* in trout fed PB diet than fed M diet has already been evidenced in a previous study [28], as well as a moderate genotype by diet interaction, which is consistent with the current conclusions: the genetic parameters obtained in the above-mentioned study allow predicting a 42% selection gain on PB diet and a 31% gain on M diet for fish selected on a PB diet (3% selection pressure), while the responses observed here are 33 and 20%, respectively. Thus, observed responses are similar to those expected, although slightly lower. On the other hand, the *BW* gain recorded in the present study contrasts with a previous selection experiment using plant-based diet substituted for fishmeal only which showed little response to selection even after four generations of selection [23]. This contrast supports the hypothesis that the lipid composition and fatty acids profile of the diet are very likely key factors of adaptation to plant-based diets. We therefore examined the whole lipid and fatty acids content of S and C groups at 291 dpf, fed the two different diets.

DM, lipid and energy content of the whole fish were modified neither by the diet nor by the selection (Table 3). Only protein content was higher in fish fed M diet (P<0.05) probably due to lower digestibility of plant-based meal. The trial also confirmed that rainbow trout is a very efficient converter of valuable fatty acids as fish fed PB diet only received 18:2n−6 and 18:3n−3 and no EPA and DHA but finally contained a large amount of these fatty acids with large benefits for human health (Table 3, Table S3). However, no changes in EPA and DHA quantities could be detected after one generation of selection under PB diet. Retention analysis associated with precise estimates of feed intake should give more information about specific abilities to retain and transform such fatty acids, in the next generations of selection.

**Table 3:** Composition of whole fish after 291 days for dry matter (DM), lipid (Lip), protein (Pt), energy (Ener), EPA (20:5n−3), DHA (22:6n−3) and statistical test for selection, diet effects and interaction.

	C-M^1^	SE	C-PB^1^	SE	S-M^1^	SE	S-PB^1^	SE	Sel^2^	Diet^2^	Sel*Diet^2^
**DM** 3	31.1^a^	0.4	31.8^a^	0.2	31.9^a^	1.3	32.0^a^	0.6	n.s.	n.s.	n.s.
**Lip** 4	12.6^a^	0.2	15.1^a^	0.1	12.4^a^	0.6	15.1^a^	0.4	n.s.	n.s.	n.s.
**Pt** 4	16.6^a^	0.3	15.2^b^	0.3	16.9^a^	0.4	15.2^b^	0.4	n.s.	***	n.s.
**Ener** 4	27.2^a^	0.6	29.3^a^	0.7	27.0^a^	0.3	29.7^a^	0.1	n.s.	n.s.	n.s.
**20:5n−3** 5	84.4^a^	4.5	6.4^b^	1.6	90.7^a^	7.4	5.8^b^	0.3	n.s.	***	n.s.
**22:6n−3** 5	114.4^a^	7.5	20.0^b^	3.2	122.2^a^	7.8	19.1^b^	0.9	n.s.	***	n.s.

1 Different superscript letters indicate significance difference between values (P<0.05).

2 *** means P<0.01,** means P<0.05, * means P<0.1, n.s. P>0.1).

3 In %.

4 In % of wet weight.

5 In mg.g lipid^−1^.

Altogether, combining the response to selection for survival and for body weight with the PB diet tested here, the cumulative biomass gain reached a maximum of +54.4% (Table 4) after 145 dpf. If the genetic gain is maintained over the next generations of selection, the selected line fed with the PB diet would reach the same biomass production as the control line fed with the M diet after approximately two generations. Taking the minimum relative *BW* gain as the reference (291 dpf), the genetic progress for biomass would still reach +43.2% and the selected strain would equal the control line fed with the M diet after approximately three generations. It should also be noted that the progress in survival rate over generations may be limited because of the structure of the trait. Thus, genetic improvement of the biomass would not be linear. Anyway, fish are excellent candidates to initiate such breeding programs since the size of broodstocks and the fertility of breeders allow high selection pressures.

**Table 4:** Observed gain (%) for body weight (*BW*), survival rate (*Su*) and total biomass (*Biom*) between selected and control lines fed on M (Gain M) or PB (Gain PB) diet and the PB-specific gain.

Trait	dpf	Gain M^1^	Gain PB^2^	PB-specific gain^3^
***BW***	60	9.2%	21.4%	12.2%
***BW***	102	20.0%	33.3%	13.3%
***BW***	145	20.3%	35.3%	15.0%
***BW***	168	16.2%	32.4%	16.2%
***BW***	193	11.3%	29.7%	18.4%
***BW***	216	10.5%	26.1%	15.6%
***BW***	236	9.4%	26.3%	16.9%
***BW***	291	7.8%	18.2%	10.5%
***BW***	312	6.2%	17.8%	11.6%
***BW***	355	3.8%	15.7%	11.9%
***Su***	145	0%	15.1%	15.1%
***Su***	193	0%	16.2%	16.2%
***Su***	291	0%	21.1%	21.1%
***Biom***	145	19.0%	54.4%	35.4%
***Biom***	193	10.0%	50.1%	40.1%
***Biom***	291	0.2%	43.2%	43.0%

1 Calculated with model (2).

2 Calculated with model (3).

3 Calculated with model (4) as the difference between Gain M and Gain PB.

When using highly substituted plant-based diets, groups of small, moribund fish were usually observed in preliminary trials [28]. This motivated a detailed analysis of the within-group distributions of weights since they provide an indication of fish health and welfare. Compared to the M diet, the PB diet was associated with higher within-group coefficients of variation (P<0.05; Fig. 1D). This elevated heterogeneity not only reflects the underlying genetic variability, but is somewhat related to a higher phenotypic instability at the individual level because of difficulties to cope with the plant-based diet, as revealed in dietary trials using isogenic trout lines [21]. Interestingly, the coefficient of variation was significantly lower in the selected line than in the control when fed the PB diet (P<0.05), suggesting that selection may have simultaneously improved the health and welfare of the selected fish. Performance homogeneity is an important trait for production, and the more homogeneous the distribution is, the more valuable the cohort will be. The beneficial effects of selection for ability to grow on plant-based diets on fish behavior and health need further investigation. Anyhow, the reduction of phenotypic variability observed in the selected line opens interesting perspectives on selection to improve farmed fish welfare.

Understanding which biological traits are targeted by diet-related selective pressures is a key issue. The selected trout line is therefore a valuable resource for carrying out investigations intended to identify underlying mechanisms of adaptation to plant-based diets. A set of processes that include sensory perception and appetite control, digestive tract morphology and physiology and metabolism will probably be involved [6]. The present trial emphasized that early development stages are determinant since much of the selection gain was recorded in the first months of life. The early ability to digest feedstuffs, to use fatty acids, feed attraction plasticity and the impact of diet changes on the nutritional quality of the product are therefore relevant features to be further investigated.

What the consumer eats is increasingly becoming a social responsibility and a political challenge. Within the context of resource limitation, the consumption of primarily carnivorous species produced by fisheries [29] or aquaculture is widely challenged. The current shift to a plant-based diet in carnivorous farmed fish may have a deleterious effect during the first stages of adaptation, and selection may be an effective tool to rapidly improve survival, growth and welfare at critical stages. The availability of products for animal feed will be a central issue in the coming years, and the challenge will be to adapt farmed fish to less standardized diets, with the increased use of plant-based materials. It should be noted that the components of the experimental PB diet were carefully chosen here for their high nutritional value and digestibility, but some of them cannot be considered as sustainable substitutes. Competition for land use and cultivation of some of the terrestrial plant-based products will raise economic, environmental and social challenges. Regardless of whether the ability of fish to adapt to plant-based diets through selection can be further improved, the ability to adapt to diets containing local and/or less sought-after products is the next important question that must be addressed. By combining new plant-based diets that are both environmentally friendly and adapted to fish nutritional requirements with breeding programs designed to enhance the ability of fish to use these alternative feed-stuffs, it would be possible to improve fish welfare and to ease the transition towards a more sustainable aquaculture production.

## Supporting Information

Table S1 **Fatty acid composition of the experimental diets.**
(DOCX)

Table S2 **Ls-means of body weight (** ***BW*** **), survival rate (** ***Su*** **) and biomass (** ***Biom*** **) after 145 days post-fertilization (dpf).**
(DOCX)

Table S3 **Mean and standard deviation of fatty acid composition (FA).**
(DOCX)
