# Supplementary material for: Selection for Adaptation to Dietary Shifts: Towards Sustainable Breeding of Carnivorous Fish
Source: PLoS One. 2012 Sep 28;7(9):e44898. doi: 10.1371/journal.pone.0044898 (PMC3460975; doi:10.1371/journal.pone.0044898)
Supplement: Table S1 — Fatty acid composition of the experimental diets. (DOCX) [file pone.0044898.s001.docx]

Table S1. Fatty acid composition of the experimental diets

| **Diets^1^** | **M1^2^** | **M2^2^** | **PB1^3^** | **PB2^3^** |
| --- | --- | --- | --- | --- |
| 10 : 0 | 1.87 | - | 1.31 | - |
| 14 : 0 | 8.15 | 8.14 | 0.30 | 0.21 |
| 15 : 0 | 0.49 | 0.44 | 0.04 | 0.03 |
| 16 : 0 | 16.71 | 16.20 | 14.18 | 12.71 |
| 17 : 0 | 0.40 | 0.45 | 0.07 | 0.15 |
| 18 : 0 | 2.32 | 2.76 | 2.53 | 2.63 |
| 20 : 0 | 0.14 | 0.13 | 0.30 | 0.34 |
| 16 : 1 | 8.23 | 7.75 | 0.26 | 0.16 |
| 18 : 1 | 11.55 | 10.52 | 37.88 | 38.69 |
| 20 : 1 | 3.60 | 1.71 | 0.66 | 0.67 |
| 22 : 1 | 5.10 | 2.33 | 0.20 | 0.24 |
| 16 : 2*n*-4 | 1.37 | 1.50 | 0.02 | - |
| 16 : 3*n*-4 | 1.53 | 2.08 | 0.06 | 0.05 |
| 16 : 4*n*-1 | 2.07 | 3.43 | - | - |
| 18 : 2*n*-6 | 2.54 | 1.67 | 22.72 | 21.67 |
| 20 : 4*n*-6 | 0.74 | 0.82 | - | - |
| 22 : 2*n*-6 | 0.23 | 0.33 | 0.21 | 0.22 |
| 18 : 3*n*-3 | 0.96 | 0.79 | 17.87 | 20.13 |
| 18 : 4*n*-3 | 2.65 | 3.13 | 0.02 | - |
| 20 : 4*n*-3 | 0.61 | 0.64 | - | - |
| 20 : 5*n*-3 | 12.88 | 16.38 | 0.15 | - |
| 21 : 5*n*-3 | 0.53 | 0.20 | - | - |
| 22 : 5*n*-3 | 1.35 | 1.74 | - | - |
| 22 : 6*n*-3 | 9.34 | 10.11 | 0.07 | - |
| Sum | 28.42 | 32.99 | 18.11 | 20.13 |

^1^ Composition given in g/100 g of total fatty acid

^2^ M1 diet was given from first feeding to 236 dpf and M2 from 236 dpf until the end of the trial to adapt protein and lipid content to the nutritional requirements of larger fish.

^3^ PB1 diet was given from first feeding to 280 dpf and PB2 from 280 dpf until the end of the trial to adapt protein and lipid content to the nutritional requirements of larger fish.
