# Supplementary material for: Selection for Adaptation to Dietary Shifts: Towards Sustainable Breeding of Carnivorous Fish
Source: PLoS One. 2012 Sep 28;7(9):e44898. doi: 10.1371/journal.pone.0044898 (PMC3460975; doi:10.1371/journal.pone.0044898)
Supplement: Table S2 — Ls-means of body weight ( BW ), survival rate ( Su ) and biomass ( Biom ) after 145 days post-fertilization (dpf). (DOCX) [file pone.0044898.s002.docx]

Table S2. Ls-means of body weight (*BW*), survival rate (*Su*) and biomass (*Biom*) after 145 days post-fertilization (dpf).

| **Trait^1^** | **Dpf** | **C-M** | **C-PB** | **S-M** | **S-PB** | **CS-M** | **SC-M** | **CS-PB** | **SC-PB** |
| --- | --- | --- | --- | --- | --- | --- | --- | --- | --- |
| ***BW*** | 145 | 6.9±0.1^a^ | 3.4±0.1^b^ | 8.3±0.1^c^ | 4.6±0.1^d^ | 7.5±0.1^c^ | 7.9±0.1^c^ | 4.0±0.1^e^ | 4.0±0.1^e^ |
| ***Su*** | 145 | 91.5±1.1^a^ | 66.9±1.1^b^ | 90.3±1.1^a^ | 77.0±1.1^c^ | 91.5±1.1^a^ | 93.7±1.1^a^ | 77.9±1.1^c^ | 74.8±1.1^c^ |
| ***Biom*** | 145 | 3.5±0.0^a^ | 1.3±0.0^b^ | 4.1±0.0^c^ | 2.0±0.0^d^ | 3.8±0.0^c^ | 4.1±0.0^c^ | 1.7±0.0^e^ | 1.6±0.0^e^ |

^1^ Standard-errors are indicated after each value and different superscript letters mean values are significantly different (P<0.05, model (1)).
