# Supplementary material for: Selection for Adaptation to Dietary Shifts: Towards Sustainable Breeding of Carnivorous Fish
Source: PLoS One. 2012 Sep 28;7(9):e44898. doi: 10.1371/journal.pone.0044898 (PMC3460975; doi:10.1371/journal.pone.0044898)
Supplement: Table S3 — Mean and standard deviation of fatty acid composition (FA). (DOCX) [file pone.0044898.s003.docx]

Table S3. Mean and standard deviation of fatty acid composition (FA)

| **FA^1^** | **C-M^2^** | **SE** | **C-PB^2^** | **SE** | **S-M^2^** | **SE** | **S-PB^2^** | **SE** | **Sel^3^** | **Diet^3^** | **Sel*Diet^3^** |
| --- | --- | --- | --- | --- | --- | --- | --- | --- | --- | --- | --- |
| **10:0** | 5.7^a^ | 2.2 | 5.3^a^ | 2.6 | 6.0^a^ | 2.4 | 5.1^a^ | 0.5 | n.s. | n.s. | n.s. |
| **12:0** | 0.9^a^ | 0.0 | 0.5^a^ | 0.1 | 1.0^a^ | 0.1 | 0.4^a^ | 0.0 | n.s. | n.s. | n.s. |
| **14:0** | 76.3^a^ | 2.9 | 6.6^b^ | 0.4 | 75.7^a^ | 2.3 | 6.5^b^ | 0.4 | n.s. | *** | n.s. |
| **15:0** | 5.1^a^ | 0.2 | 0.9^b^ | 0.0 | 4.9^a^ | 0.2 | 0.9^b^ | 0.0 | n.s. | *** | n.s. |
| **16:0** | 193.3^a^ | 7.2 | 154.6^ab^ | 4.2 | 187.9^ab^ | 2.9 | 151.5^b^ | 2.6 | n.s. | n.s. | n.s. |
| **17:0** | 3.8^a^ | 0.1 | 1.4^b^ | 0.2 | 4.0^a^ | 0.2 | 1.4^b^ | 0.1 | n.s. | *** | n.s. |
| **18:0** | 32.6^a^ | 0.7 | 34.2^b^ | 0.7 | 32.0^a^ | 0.8 | 35.7^b^ | 1.2 | n.s. | *** | ** |
| **20:0** | 1.1^a^ | 0.1 | 1.8^b^ | 0.2 | 0.5^c^ | 0.2 | 2.0^b^ | 0.1 | n.s. | n.s. | *** |
| **Saturates** | 318.7^a^ | 10.9 | 205.3^b^ | 3.9 | 312.0^a^ | 4.9 | 203.5^b^ | 3.1 | n.s. | *** | n.s. |
| **14:1** | 0.6 | 0.1 | - | - | 0.6 | 0.0 | - | - | - | - | - |
| **15:1** | - | - | - | - | 0.6 | 0.2 | - | - | - | - | - |
| **16:1** | 89.6^a^ | 3.9 | 24.6^b^ | 1.1 | 88.6^a^ | 2.6 | 23.5^b^ | 2.0 | n.s. | *** | n.s. |
| **17:1** | 0.5 | 0.1 | 0.^5^ | 0.1 | - | - | 0.4 | 0.0 | - | - | - |
| **18:1** | 160.0^a^ | 5.2 | 380.0^b^ | 14.8 | 155.6^a^ | 6.5 | 391.7^b^ | 1.7 | n.s. | *** | n.s. |
| **20:1** | 27.4^a^ | 1.1 | 9.9^ab^ | 0.8 | 25.5^ab^ | 2.3 | 10.2^b^ | 0.8 | n.s. | n.s. | n.s. |
| **22:1** | 25.0^a^ | 2.2 | 1.7^ab^ | 0.4 | 22.6^ab^ | 3.4 | 1.7^b^ | 0.2 | n.s. | n.s. | n.s. |
| **MUFA** | 303.2^a^ | 7.4 | 416.6^b^ | 14.8 | 293.5^a^ | 12.3 | 427.4^b^ | 2.3 | n.s. | ** | n.s. |
| **16:2 n-4** | 11.7 | 0.3 | - | - | 12.0 | 0.5 | - | - | - | - | - |
| **16:3 n-4** | 11.3^a^ | 0.5 | 0.8^b^ | 0.3 | 11.9^a^ | 0.7 | 0.8^b^ | 0.0 | n.s. | *** | n.s. |
| **16:4 n-1** | 11.1^a^ | 0.6 | 0.7^b^ | 0.4 | 12.3^c^ | 1.2 | 0.5^b^ | 0.1 | n.s. | *** | n.s. |
| **18:2 n-6** | 23.9^a^ | 0.4 | 178.0^b^ | 2.9 | 24.1^a^ | 0.6 | 178.6^b^ | 3.1 | n.s. | *** | n.s. |
| **18:3 n-6** | 1.9^a^ | 0.2 | 13.5^b^ | 1.9 | 1.8^a^ | 0.5 | 12.8^b^ | 0.8 | n.s. | ** | n.s. |
| **20:2 n-6** | 1.9^a^ | 0.1 | 4.2^a^ | 1.9 | 1.8^a^ | 0.2 | 4.3^a^ | 2.0 | n.s. | n.s. | n.s. |
| **20:3 n-6** | 1.7^a^ | 0.4 | 8.8^b^ | 1.0 | 1.7^a^ | 0.5 | 8.4^b^ | 0.6 | n.s. | *** | n.s. |
| **20:4 n-6** | 7.2^a^ | 0.4 | 5.9^a^ | 1.7 | 7.7^a^ | 0.7 | 5.6^a^ | 0.5 | n.s. | n.s. | n.s. |
| **22:2 n-6** | 2.6^a^ | 0.3 | 3.2^a^ | 1.3 | 3.4^a^ | 0.7 | 2.6^a^ | 0.3 | n.s. | n.s. | n.s. |
| **n-6 PUFA** | 39.2^a^ | 0.9 | 213.6^b^ | 5.6 | 40.5^a^ | 1.2 | 212.1^b^ | 3.3 | n.s. | *** | n.s. |
| **18:3 n-3** | 7.5^a^ | 0.1 | 87.2^b^ | 1.2 | 7.7^a^ | 0.2 | 83.4^b^ | 1.5 | n.s. | *** | ** |
| **18:4 n-3** | 17.3^a^ | 0.5 | 28.6^a^ | 4.5 | 18.3^a^ | 1.0 | 25.9^a^ | 1.6 | n.s. | n.s. | n.s. |
| **20:3 n-3** | 0.9^a^ | 0.1 | 1.7^a^ | 0.2 | 0.8^a^ | 0.1 | 1.6^a^ | 0.2 | n.s. | n.s. | n.s. |
| **20:4 n-3** | 9.1^a^ | 0.5 | 3.9^b^ | 0.4 | 9.2^a^ | 0.3 | 3.6^b^ | 0.5 | n.s. | ** | n.s. |
| **20:5 n-3** | 84.4^a^ | 4.5 | 6.4^b^ | 1.6 | 90.7^a^ | 7.4 | 5.8^b^ | 0.3 | n.s. | *** | n.s. |
| **21:5 n-3** | 5.1^a^ | 0.4 | - | - | 5.2^a^ | 0.2 | 0.3^b^ | 0.1 | n.s. | *** | n.s. |
| **22:5 n-3** | 22.2^a^ | 1.3 | 2.3^b^ | 0.2 | 22.6^a^ | 0.9 | 2.5^b^ | 0.2 | n.s. | *** | n.s. |
| **22:6 n-3** | 114.4^a^ | 7.5 | 20.0^b^ | 3.2 | 122.2^a^ | 7.8 | 19.1^b^ | 0.9 | n.s. | *** | n.s. |
| **n-3 PUFA** | 260.8^a^ | 13.5 | 149.8^b^ | 8.5 | 276.8^a^ | 15.6 | 142.3^b^ | 2.3 | n.s. | ** | n.s. |

^1^ In mg.g^-1^ lipid

^2^ Different superscript letters indicate significance difference between values (P<0.05) and are based on within-diet comparison when sel*diet interaction was significant.

^3^ *** means P<0.01,** means P<0.05, * means P<0.1, n.s. P>0.1).
